# Paediatric medicine issues and gaps from healthcare workers point of view: survey results and a narrative review from the global accelerator for paediatric formulations project

**DOI:** 10.3389/fphar.2023.1200848

**Published:** 2023-07-17

**Authors:** Elisa Barbieri, Chiara Minotti, Sara Cavagnis, Carlo Giaquinto, Bernadette Cappello, Martina Penazzato, Marc Lallemant

**Affiliations:** ^1^ Division of Pediatric Infectious Diseases, Department of Women’s and Children’s Health, University of Padova, Padova, Italy; ^2^ Penta—Child Health Research, Padova, Italy; ^3^ Department of Health Products Policy and Standards, World Health Organization, Geneva, Switzerland; ^4^ WHO Research for Health Department, World Health Organization, Geneva, Switzerland; ^5^ Faculty of Associated Medical Sciences, Chiang Mai University, Chiang Mai, Thailand

**Keywords:** medicine access, essential medicines, children, paediatric formulation, survey

## Abstract

The WHO Model List of Essential Medicines for Children (EMLc) has not been systematically revised in the last few years. We conducted a survey addressed to healthcare professionals prescribing, preparing, or administering medicines to children and a narrative review to identify problematic paediatric formulations or missing medicines in all therapeutic fields to inform the review of the EMLc in 2023. A total of 285 physicians (63%), 28 nurses (6%) and 142 pharmacists (31%), mostly working in the hospital setting, reported at least one problematic medicine. 290 medicines were reported as missing (completely or the child-appropriate formulation). The top three most mentioned were ciprofloxacin together with phenobarbital and omeprazole. 387 medicines were reported as problematic (34% were oral liquid formulations, 34% tablets, 18% parenteral preparations. Mostly of the products were antibacterials (27%), cardiovascular medicines (11%) and antivirals (11%). The obtained responses show the perspective of healthcare workers working around the world, particularly in the European region (25%), in the African region (24%), and in the Region of the Americas (19%), with limited representation from Northern Africa and the Middle East. Our results need to be analysed with the outputs of other ongoing works before specific products can enter the WHO-hosted Global Accelerator for Paediatric formulations network prioritisation process. Efforts to develop appropriate formulations for children should be accelerated so that the uncertainties associated with off-label drug preparation and use are minimised, and therapeutic benefits are optimised.

## Background

As defined by the World Health Organization (WHO), “Essential medicines” for adults and children are those that satisfy the priority healthcare needs of a population. The goal is to ensure that quality assured medicines are always available in appropriate dosage forms within functioning health systems, and at affordable prices. Today, approximately 140 countries base their drug procurement on the WHO Model List of Essential Medicines for Children (EMLc) (WHO, 2023a).

The current EMLc includes more than 300 medicines but multiple products of major clinical importance are missing from the list. Reasons for their absence include lack of proper evaluation, delayed paediatric development of products available for adults, approval for special uses only, or inappropriate formulations for children, especially for neonates and young infants (O’Brien et al., 2019; Walsh et al., 2021; Moore-Hepburn and Rieder, 2022).

The Global Accelerator for Paediatric Formulations (GAP-f) is a WHO Network hosted within the Research for Health Department of the Science Division. It has been created to respond to the paediatric treatment gap by accelerating the pharmaceutical development, registration, and procurement of missing formulations. GAP-f has recently started to identify and prioritise missing paediatric formulations for the WHO Model List of EMLc. This scoping exercise will enable it to assess the appropriateness of existing paediatric formulations and to identify overall gaps. This process will feed into future EMLc revisions and inform inclusion of additional missing formulations in the GAP-f portfolio. This will ensure that, in the long-term, children in low- and middle-income countries will be able to access a range of essential medicines in appropriate formulations.

To date, findings from the literature confirm there is a global need for child-appropriate formulations for different age categories (Walsh et al., 2022). Indeed, gaps exist for low- and high-resource contexts alike (Ivanovska et al., 2017; Walsh et al., 2019; Delmoral-Sanchez et al., 2020; Orubu et al., 2022).

To contribute to the upcoming review of the EMLc by the WHO, we conducted a survey qualitative study addressed to healthcare professionals actually prescribing, preparing, or administering medicines to children, and a narrative review in order to identify problematic paediatric formulations or missing medicines for children in all therapeutic fields.

### Material and methods

#### Survey design

We created and launched an online survey using the REDCap platform to investigate different aspects of children’s medicine formulations, including problematic and missing formulations, identified as such by the respondents themselves based on their professional experience. The different issues were discussed based on the specialisation of healthcare professionals: physicians, nurses, and pharmacists.

The survey explored.

1. Child acceptability (physicians and nurses).
2. Ease of use for children and caregivers (physicians and nurses).
3. Off-label use (physicians and pharmacists).
4. Extemporaneous preparation (pharmacists only).
5. Complexity of reconstitution and stability issues (pharmacists only).
6. Dosing and safety issues (physicians only).
7. Use in special situations or fragile populations (physicians and pharmacists).
8. Formulations missing for any reason (all).

A complete description of the survey is included in the Supplementary Material.

#### Targeting of respondents and distribution of survey materials

Prospective respondents comprised: general health practitioners taking care of children (family and general practitioners/paediatricians, and pharmacists), and experts representing relevant paediatric subspecialties.

There was no specific target for the number of respondents, but the aim was to cover a broad geographical area and all therapeutic fields.

A list of all the scientific national and sub-national scientific societies listed members of the International Society of Paediatrics, of the International Council of Nurses, of the World Organization of Family Doctors and the International Pharmaceutical Federation was created, and electronic contacts were extracted from websites and publicly available information by two authors (E.B. and S.C.). In addition, electronic contacts of international scientific societies and networks involved in paediatric research were included in the list. The complete list of the contacted societies is provided in the Supplementary Material.

The objectives of the project and the scope of the survey were presented in a newsletter written in English, French, Spanish, Arabic, and Portuguese, that provided a direct link to the REDCap platform that hosted the survey. A guide on how to use the automatic translation option in Google Chrome was created and added as a link to the newsletter and to the survey instructions, to facilitate the completion of the survey by respondents from all Countries using languages other than English.

The survey went online on 6th July 2021 and was distributed to the scientific and medical societies that had previously registered via the newsletter.

If a Society did not respond, a reminder was sent after 15 days.

#### Incomplete surveys and data validation

Respondents who did not complete the survey in full, but who provided a valid email address, received an automatic reminder to complete the survey within 10 days. A final reminder was sent 3 days before the closing date, 30th September 2021.

Two blinded investigators (E.B. and S.C) excluded from the analysis any survey responses in which valid personal or country information was not provided.

Free text fields were manually validated (E.B. and S.C.); if containing information that could have been included in structured fields, they were incorporated in the appropriate fields.

Medicines were classified based on the WHO EMLc classification system (C.M. and E.B.); medicines that were not listed in the EMLc were classified in the most suitable group.

#### Data analysis

The data were summarised descriptively as numbers and percentages and stratified by medicine class, active product ingredient and specific pharmaceutical forms, where possible. Microsoft Excel was used to create the descriptive tables and R Foundation Statistical Program (version 4.1.1) and R Studio were used to create the global maps.

#### Role of the funding source

The funder of the study had no role in study design, data collection, data analysis, data interpretation, or the writing of the report.

### Main results

A total of 1,326 people logged on to the RedCAP survey website, 925 started the survey providing demographic data, and 455 responded to the two main survey questions, listing the products or formulations found most problematic and/or missing. The flow chart of respondents with reasons for exclusion is reported in Supplementary Figure S1 in the Supplementary Material.

**FIGURE 1:**
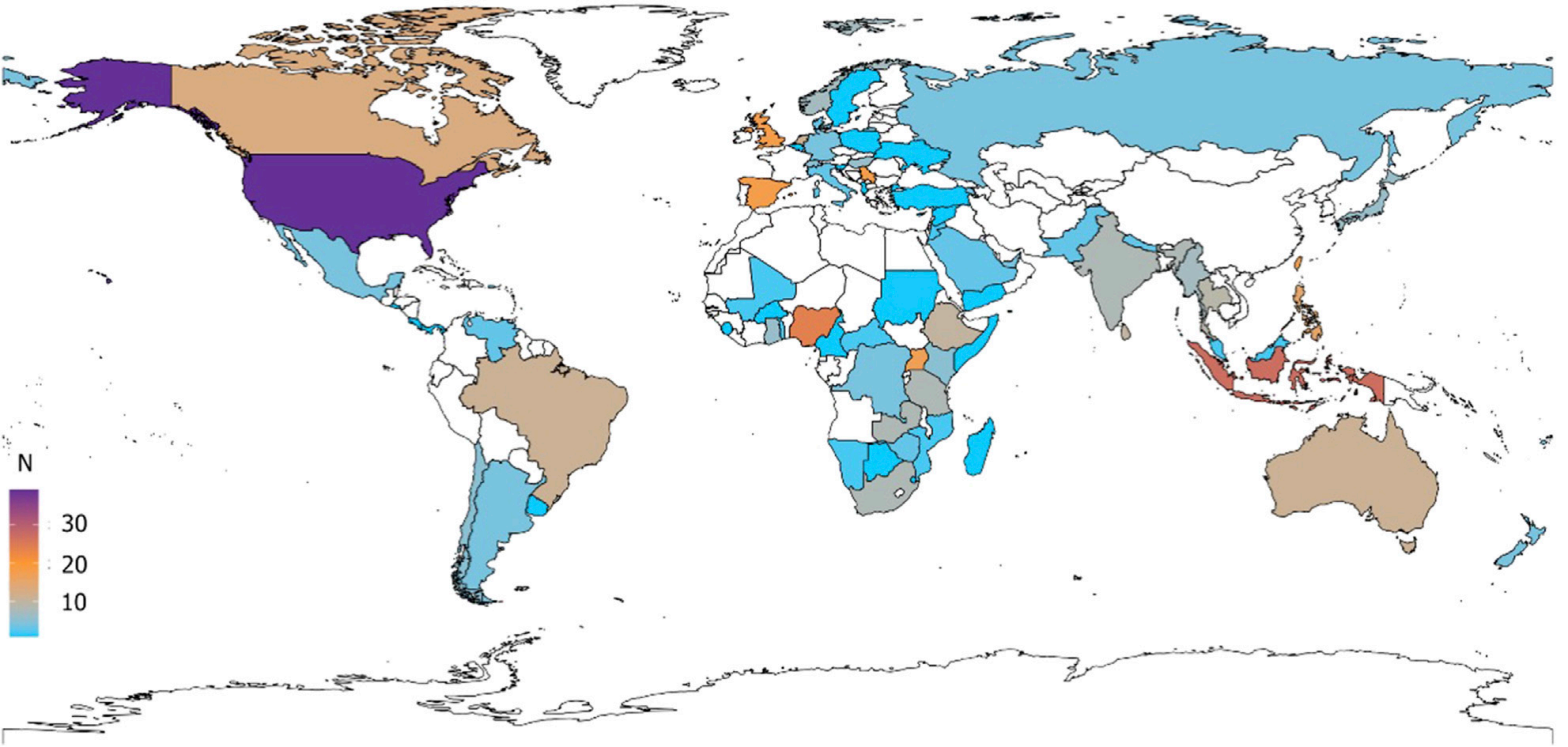
Map of the countries where the respondents stated they practised.

Of the 455 respondents, 114 (25%) practice in the European region, 110 (24%) in the African region, 85 in the Region of the Americas (19%), 65 (14%) in the Western Pacific region, 64 (14%) in the South-East Asian Region, and 17 (4%) in the Eastern-Mediterranean region. Figure 1 is the geographical representation of overall respondents and the maps stratified by healthcare professional speciality can be found in Supplementary Figure S2 the Supplementary Material.

A total of 285 physicians (63%), 28 nurses (6%) and 142 pharmacists (31%) reported at least one problematic medicine. Of these, 237 physicians (83%), 21 nurses (75%) and 112 pharmacists (79%) worked in a hospital setting. All paediatric subspecialties were represented, half were general paediatrics. Emergency and intensive care medicine, infectious diseases, and neonatology were well represented. Haemato-oncology was widely covered among health workers working in hospitals, nutrition among nurses working in primary care units and allergy among pharmacists working in primary care as well (Supplementary Table S1 in the Supplementary Material).

#### Missing pharmaceutical products

Overall, 290 medicines were reported as missing (either completely, either the child-appropriate formulation) from a total of 794 pharmaceutical forms mentioned in the survey.

Ciprofloxacin was the most cited product (18 times) together with phenobarbital (Table 1). Ciprofloxacin was reported to be available only as an adult formulation in Australia, Chile, Nigeria, Philippines, Portugal, Serbia, Uganda, and Zambia. When stratifying by pharmaceutical form, ciprofloxacin was missing as oral liquid and tablet formulations; paediatric tablets were reported missing by respondents working in Australia, Nigeria, Uganda and Switzerland (Table 1).

**TABLE 1:** Missing pharmaceutical products (N > =10) for paediatric care according to physicians, nurses, and pharmacists.

**Medicine**	**N tot**	**N countries**	**Only Available As Adult Formulation**	**Not Marketed In The Country**	**Only Available In The Private Sector**	**Overly Expensive**	**Not In The Drug Formulary**	**Shortages**	**Other**	**NA**
*Ciprofloxacin*	18	9	Australia Chile Nigeria Philippines Portugal Serbia Uganda Zambia	Chile Philippines					Switzerland	
*Phenobarbital*	18	12	Ethiopia Germany Indonesia Myanmar Philippines Serbia Zambia	Mexico Netherlands Serbia Thailand Zambia		Zambia	South Africa Zambia	Indonesia Philippines South Africa Zambia	Argentina South Africa	
*Omeprazole*	16	14	Brazil Chile Hungary Indonesia Philippines Saudi Arabia Thailand Zambia	Chile Netherlands Spain Sri Lanka Zambia	Brazil	Australia Germany	Fiji Germany Zambia	Zambia	Australia Germany	
*Abacavir + dolutegravir + lamivudine*	15	10	Australia Brazil Chile Congo Kinshasa Indonesia Uganda United Kingdom	Botswana Chile Indonesia South Africa			Zambia		Indonesia	
*Furosemide*	15	9	Brazil Ethiopia Myanmar Nigeria Serbia Switzerland Tanzania Zimbabwe	Philippines Serbia			Rwanda	Ethiopia Rwanda		
*Levothyroxine*	13	9	Canada Nigeria Palestinian Territory Portugal Taiwan United Kingdom United States	Canada New Zealand Palestinian Territory Taiwan United Kingdom		United States			Portugal United States	
*Caffeine*	12	9	Tanzania	Nepal Tanzania United Arab Emirates Venezuela	Ghana	Myanmar United States	Argentina Tanzania Thailand United States	Nepal	Argentina Thailand	
*Prednisolone*	12	10	Ghana Hungary Nigeria Norway Portugal United Arab Emirates Zambia Zimbabwe	Albania Nigeria Zimbabwe	Zimbabwe	Zimbabwe				Sri Lanka
*Clindamycin*	11	8	Australia Ethiopia Nigeria Portugal	Argentina Spain United States				Switzerland	Switzerland	
*Clonidine*	10	7	Australia Japan Norway United States	Canada Netherlands						Sri Lanka
*Tacrolimus*	10	9	Japan Puerto Rico Taiwan United Kingdom	United Kingdom		Indonesia United Kingdom	Taiwan Zambia	Netherlands Sri Lanka	Japan	Sri Lanka
*Valproic acid*	10	9	Myanmar Tanzania Germany	Indonesia		Fiji Ghana Indonesia Nigeria Sri Lanka	Ghana	Fiji Jordan Sri Lanka		

Paediatric pharmaceutical forms (oral liquid and parenteral preparations) of phenobarbital were reported as missing, but available in many countries as adult formulations (i.e., Ethiopia, Germany, Indonesia, Myanmar, Philippines, Serbia, Zambia) Moreover, phenobarbital shortages were reported for Indonesia, Philippines, South Africa and Zambia (Table 1).

The paediatric pharmaceutical form of omeprazole (especially suppositories and capsules) was reported as missing 16 times in 14 countries including high income countries such as the Netherlands, Germany, South Africa, Australia, Chile and Argentina. In Table 1 are reported the pharmaceutical products for paediatric care most mentioned as missing. Complete data are reported in Supplementary Tables S2, S3 in the Supplementary Material.

#### Pharmaceutical formulations considered most problematic

Overall, feedback was received on 387 medicines, for a total of 609 problematic pharmaceutical forms (34% were oral liquid formulations, 34% tablets, 18% parenteral preparations, 8% capsules and the rest a variety of pharmaceutical forms). Most of the products were antibacterials (27%), cardiovascular medicines (11%) and antivirals (11%).

The medicines that were mentioned most frequently as problematic were the liquid oral form of lopinavir/ritonavir reported (LPV/r) 27 times, followed by amoxicillin and clavulanic acid (AMC) in liquid oral form (23 times) and cefuroxime in oral liquid form (21 times). The liquid oral form of LPV/r was considered to be the most problematic pharmaceutical form by all healthcare specialists, mostly due to its poor acceptability (in 78% of cases). For salbutamol, the problem was mainly related to poor usability (in 58% of cases).

Because of the large number of pharmaceutical formulations reported as problematic, we focused on the three most frequently mentioned products for the class of medicines for which more than 50 products were mentioned (Table 2, and Supplementary Table S4 in the Supplementary Material). Complete data can be found in Supplementary Table S5 in the Supplementary Material.

**TABLE 2:** Top three most mentioned products within each class of medicine that received at least 50 responses, considered most problematic for physicians, nurses, and pharmacists, stratified by pharmaceutical form [Legend: (*) Physicians, (**) Pharmacists, (^) Physicians and nurses, (^ ^) Physicians and pharmacists].

**Class**	**Medicine**	**Pharmaceutical form**	**N**	Dosing/Safety * N (%)	Extemporaneous preparation** **N (%)**	Reconstitution and stability issue** **N (%)**	**Acceptability ^** **N (%)**	**Usability^** **N (%)**	**Off label ^ ^** **N (%)**	**Special use ^ ^** **N (%)**
Anticonvulsants/antiepileptics	phenobarbital	tablet	10	-	5 (50·0)	3 (30·0)	-	2 (20·0)	1 (10·0)	3 (30·0)
	clobazam	tablet	6	-	1 (16·7)	-	3 (50·0)	1 (16·7)	-	1 (16·7)
	valproic acid	capsule	5	2 (40·0)	1 (20·0)	1 (20·0)	-	1 (20·0)	1 (20·0)	-
Anti-infective - antibacterials	Amoxicillin + clavulanic acid	oral liquid	23	3 (13·0)	-	7 (30·4)	6 (26·1)	6 (26·1)	-	-
	cefuroxime	oral liquid	21	1 (4·8)	-	1 (4·8)	17 (81·0)	2 (9·5)	-	-
	amoxicillin	oral liquid	15	3 (20·0)	-	2 (13·3)	4 (26·7)	5 (33·3)	2 (13·3)	2 (13·3)
Anti-infective - antivirals	Abacavir + dolutegravir + lamivudine	tablet	14	5 (35·7)	1 (7·1)	2 (14·3)	3 (21·4)	3 (21·4)	2 (14·3)	3 (21·4)
	Lopinavir + ritonavir	oral liquid	27	1 (3·7)	1 (3·7)	3 (11·1)	21 (77·8)	6 (22·2)	-	3 (11·1)
	Lopinavir + ritonavir	tablet	12	2 (16·7)	-	-	8 (66·7)	4 (33·3)	-	1 (8·3)
Cardiovascular medicines	furosemide	tablet	17	7 (41·2)	4 (23·5)	1 (5·9)	4 (23·5)	5 (29·4)	1 (5·9)	1 (5·9)
	digoxin	tablet	13	7 (53·8)	4 (30·8)	1 (7·7)	3 (23·1)	4 (30·8)	2 (15·4)	1 (7·7)
	captopril	tablet	12	1 (8·3)	5 (41·7)	1 (8·3)	4 (33·3)	3 (25·0)	2 (16·7)	-
Gastrointestinal medicines	omeprazole	oral liquid	17	2 (11·8)	7 (41·2)	7 (41·2)	3 (17·6)	4 (23·5)	2 (11·8)	2 (11·8)
	omeprazole	capsule	12	3 (25·0)	3 (25·0)	3 (25·0)	5 (41·7)	5 (41·7)	3 (25·0)	2 (16·7)
	omeprazole	tablet	8	1 (12·5)	1 (12·5)	1 (12·5)	2 (25·0)	3 (37·5)	1 (12·5)	-
Immunomodulators and antineoplastics	tacrolimus	oral liquid	6	1 (16·7)	5 (83·3)	4 (66·7)	-	-	1 (16·7)	1 (16·7)
	mercaptopurine	tablet	5	-	1 (20·0)	1 (20·0)	1 (20·0)	-	-	2 (40·0)
	methylprednisolone	tablet	5	-	-	-	5 (100)	-	-	-
Medicine acting on the respiratory tract	salbutamol	preparation for inhalation	19	5 (26·3)	-	-	3 (15·8)	11 (57·9)	1 (5·3)	3 (15·8)
	aminophylline	parenteral preparation	6	2 (33·3)	-	-	1 (16·7)	2 (33·3)	2 (33·3)	1 (16·7)
	budesonide	preparation for inhalation	6	-	-	1 (16·7)	2 (33·3)	3 (50·0)	1 (16·7)	-
Medicines affecting the blood	hydroxyurea	capsule	10	4 (40·0)	4 (40·0)	1 (10·0)	2 (20·0)	2 (20·0)	1 (10·0)	1 (10·0)
	ferrous sulphate anhydrous	oral liquid	6	-	1 (16·7)	1 (16·7)	4 (66·7)	1 (16·7)	-	1 (16·7)
	enoxaparin	parenteral preparation	4	-	2 (50·0)	2 (50·0)	-	-	1 (25·0)	-
Medicines for pain and palliative care	acetaminophen	oral liquid	12	7 (58·3)	-	-	7 (58·3)	2 (16·7)	-	2 (16·7)
	acetylsalicylic acid	tablet	8	1 (12·5)	4 (50·0)	3 (37·5)	-	-	2 (25·0)	2 (25·0)
	diazepam	rectal preparation	7	3 (42·9)	1 (14·3)	-	-	-	2 (28·6)	1 (14·3)

## Discussion

Our study provided insights into the existing gaps in the development and usage of paediatric medicines. Through the analysis of responses from healthcare professionals, we identified areas that require further research and development. This information can play a crucial role in guiding research institutions and funding agencies to prioritize specific areas in paediatric drug development and optimize the allocation of resources. Additionally, the findings from our survey have the potential to enhance the safety and efficacy profiles of paediatric medications. By understanding the challenges faced by healthcare workers who care for children, regulatory agencies and research institutions can work together to address formulation issues, dosing challenges, and other concerns related to paediatric drug development. This collaborative effort can lead to the creation of more appropriate and effective paediatric dosage forms and treatment options.

The findings of our study were presented during a virtual consultation meeting with international experts in paediatric medications and other stakeholders convened by the WHO Essential Medicines team in November 2022. They complemented the findings of other analyses of the availability and suitability of existing products listed in the WHO Model List of Essential Medicines for Children. One of the aims of this consultation was to identify suboptimal formulations that should be removed from the EMLc and to identify formulations used in countries that may be valuable additions. The output was a submission to the April 2023 meeting of the WHO Expert Committee on Selection and Use of Essential Medicines as part of the biennial update of the WHO Model Lists, proposing changes to the list accordingly (WHO, 2023b). As a result, suboptimal or unavailable formulations of 17 medicines were proposed for removal, and new, age-appropriate formulations of 45 medicines were proposed for addition. The Expert Committee’s recommendations and updated EMLc are expected to be published in July 2023. The consultation also served to verify identified formulation gaps in essential medicines for children, which will be taken forward in GAP-f prioritization processes for accelerated development.

After the publication, the updated version of the list will be actively disseminated to promote the revision of national essential medicine lists. Additionally, monitoring the adoption and adaptation of the WHO Model List of Essential Medicines for Children, as well as the use, safety, and effectiveness of newly introduced products, will provide systematic feedback for the global prioritization process across different therapeutic areas. This process will ensure that the best available formulations are effectively prioritized on a global and country level.

Overall, 290 of 794 medicines were reported as missing in the paediatric form. Ciprofloxacin and phenobarbital were the products most mentioned as missing.

Indeed, anti-infective agents account for 15% of all drug shortages, significantly impacting the selection of the antimicrobial for the treatment. In a study conducted in the US analysing data from 2001 to 2013, fluoroquinolones were ranked fifth among the antibacterials with the highest shortage episodes in months. The most common reason for shortage included manufacturing problems and insufficient supply–demand ratio (Quadri et al., 2015). Alternative treatments may not be as familiar to clinicians and thus may lead to medication errors and adverse outcomes (in up to 20% of cases of treatment switch).

The management of seizures and status epilepticus is centred on urgent administration of appropriately dosed antiepileptics, including phenobarbital. In the intensive care settings, the treatment usually begins with intravenous IV) formulations given the rapid absorption and decreased time to achieve target serum concentrations compared to the oral formulations. Conversion from IV to oral of antiepileptics that are not highly protein bound is reasonable in patients whose seizures have abated, however drug-drug and drug-food interactions should be considered. Still, alternative treatments and methods of administration are needed in case of shortages (including the IV diluent). For example, the smart infusion pump syringe module has been proposed as an alternative administration for the IV phenobarbital, but the maximum volume to be infused should not exceed 60 mL (Society of Critical care Medicine, 2022). Moreover, in October 2022 following a rise in hand-foot-mouth disease (HFMD) in Vietnam, different hospitals updated the guidelines for the prevention and treatment of HFMD-related seizures accounting for phenobarbital shortages. However, other sedatives were reported to be less effective than phenobarbital and to cause respiratory failure if used for a long time (Vietnamnews, 2023).

Omeprazole is an over-the-counter medication, with a growing market size, especially in Europe and North America, following the increase in gastrointestinal disorders and use of cardiovascular medications. On the contrary, the market for the oral formulation (mainly for infants and children) is quite limited. Moreover, compounding an oral pharmaceutical form sometimes poses a challenge given the unsuitability of excipients or commercially available vehicles for the paediatric population. For this reason, alternative formulations are being studied and proposed, such as rectal suppositories, which seem promising. (Bestebreurtje et al., 2020a; Bestebreurtje et al., 2020b).

It is important that the paediatric formulations offer flexibility for dose adjustment, while remaining within effective therapeutic range. Oral liquid formulations have major disadvantages such as chemical, physical or microbial instability, taste issues, need for refrigerated storage conditions and lack of controlled release properties. WHO now recommends that, where available, dispersible tablets should be chosen above suspensions due to advantages in dosing, stability, storage, cost and transportation, especially in LMICs (Angwa et al., 2020; Vallet et al., 2021; Parrish et al., 2022; WHO, 2023a).

Below, we narratively review and discuss the most frequently mentioned products reported as problematic by class.

### Anti-infectives—antibacterials

Oral liquid formulations of AMC cefuroxime and amoxicillin were the top three antibacterial formulations most often reported as problematic (in 23,21 and 15 cases). AMC oral liquid was reported as problematic because of stability issues after the reconstitution of the formulation in 30% of cases (mostly related to reduced access to clean water for reconstitution and rapid deterioration of the reconstituted formulation), acceptability by children and caregiver usability in 26% of cases respectively (mostly because of formulation taste/after taste, formulation texture and high volume of liquid for a single dose as well as the need to be stored at a specific temperature) and concerns related to dosing and safety (13% of cases). Literature confirms the reported stability issues for AMC, with clavulanic acid not being as heat-stable as amoxicillin in either IV or reconstituted oral suspension forms (Mack et al., 2021). At 2°C–8°C, Mehta et al. observed a 10% degradation after 7 days, while at 20°C or 27°C–29°C ambient temperature, degradation rose to 40% or 45% in the same time frame, respectively (Mehta et al., 1994). In terms of acceptability, Angelilli et al. determined palatability using a single-blind comparison of four flavoured antimicrobial agents (azithromycin, cherry flavoured; cefprozil, bubble gum flavoured; cefixime, strawberry flavoured and AMC, banana flavoured); the palatability score for AMC was lowest (Angelilli et al., 2000). Llor et al. reported a very low rate of compliance with thrice-daily AMC regimen, but better compliance with a twice-daily regimen (Llor et al., 2012).

Cefuroxime oral liquid issues were mostly related to children’s reduced acceptability (81% of cases) because of unpleasant taste/after taste while different respondents reported that the oral liquid formulation of amoxicillin alone was complex to prepare.

Poor acceptability of cefuroxime has been reported by others: according to Steele et al., evaluating palatability and compliance issues of several antibiotic suspensions, cefuroxime ranked last for palatability (Steele et al., 2006). Considering other variables, such as costs, duration of therapy, and dosing intervals, it ranked second to last, before AMC.

The main issues with amoxicillin oral liquid formulations were usability and acceptability, due to palatability and reconstitution issues. As described in several studies, palatability can even affect prescribing practice, with other molecules, such as cefdinir, being preferred for taste and smell. However, amoxicillin is often considered better than azithromycin (Holas et al., 2005; Bradshaw et al., 2016).

### Cardiovascular medicines

Tablet formulations of furosemide, digoxin and captopril were the top three cardiovascular medicines most often reported as problematic (in 17,13 and 12 cases).

Furosemide and digoxin tablets were reported to have complexities in dosing (in 41% and 54% of cases), generating frequent dosing errors, and to be frequently prepared extemporaneously through the modification of adult products. The main reported issues in the literature with furosemide tablets are dosing and safety, poor acceptability, need for extemporaneous preparation or manipulation of adult tablets through splitting or crushing (Pacifici, 2012; Freerks et al., 2020). However, adult product modifications often influence dose accuracy, on the integrity of coatings (if any), on the stability of the active pharmaceutical ingredients and on the taste. Extemporaneous liquid compounded forms are also suboptimal, as they contain inappropriate solvents or excipients, have palatability issues, or, if the active pharmaceutical ingredient is suspended, present a risk of dosing mistakes because of non-homogeneity. To overcome such issues, paediatric immediate release mini-tablet formulations of furosemide are in development (Freerks et al., 2020; Lafeber et al., 2021).

Digoxin requires therapeutic drug monitoring because of its narrow therapeutic index. Indeed, numerous studies in the literature report high interindividual dose and exposure variability of this medicine (Moffett et al., 2016; Abdel Jalil et al., 2020).

Captopril requires extemporaneous preparation from the adult formulations because the tablet pharmaceutical forms are not well accepted by children and their dissolution in a liquid is complicated for caregivers. To date, captopril in the UK is only licensed as tablets, with many children having difficulties swallowing them. Tablets are crushed and dissolved in water, which may determine dose inaccuracies as well as altered absorption (Mulla et al., 2007). Captopril in liquid formulation, where available, is known to be potentially unstable. In some settings a licensed liquid formulation is available, in others it is available only as an extemporaneous preparation.

### Anti-infectives—antivirals

Oral liquid and tablet formulations of LPV/r as well as tablet formulation of abacavir, dolutegravir/lamivudine were most often reported as problematic. LPV/r oral liquid formulations and tablets were reported to be very poorly accepted by children in 78% and 67% of cases, respectively; for LPV/r it was related to very bitter taste and lingering aftertaste of the liquid formulations, and the fact that LPV/r liquids are formulated separately and are complex to administer (Best et al., 2011). In the second case, issues relate to the size and taste of the tablet if broken. Acceptability was the most often encountered issue in studies on LPV/r. Tablet administration, to be swallowed whole, with or after meals, may enhance gastrointestinal tolerability, with a decrease in diarrhoea, nausea and vomiting, and bad taste in the mouth. In the literature it was found that crushed tablets are slowly and erratically absorbed, and result in significantly reduced AUC, Cmax, and Ctrough compared with swallowing the whole tablet. Following this reduction by 5%–75% in the AUC, a dose modification of the crushed tablets would not overcome reduced absorption (Puthanakit et al., 2010). Notably new solid pellets or granules formulations have been developed that start reaching the market, at least in resource limited settings where they are produced by generic companies (e.g., Viatris, Cipla) under licence from the originator company.

Lack of safety data and pharmacokinetic evidence for the different dosing regimens for the fixed dose combination of abacavir, dolutegravir/lamivudine was the most reported issue, raising the need to for the medicine to be diluted to enable the correct dose to be measured.

Dosing, bioavailability and safety were reported as main issues with abacavir and dolutegravir/lamivudine preparations (Zhao et al., 2013). Optimal paediatric formulations are needed, especially fixed-dose combinations, to ensure correct dosing, easier swallowing and palatability (Singh et al., 2022). These fixed-dose combinations with appropriate paediatric dosing have also been developed that start reaching the market (Brooks et al., 2019; Clinicalinfo.hiv, 2023).

### Anticonvulsants/antiepileptics

Solid oral formulation of phenobarbital (tablets), clobazam (tablets) and valproic acid (capsules) were the antiepileptic medicines most often reported as problematic (in ten, six and five cases); for all, the requirement for extemporaneous preparation of paediatric formulations from adult formulations (in half of the cases for phenobarbital) was reported.

Differently from our findings, the main issues emerging from the literature about phenobarbital relate mainly to its IV use in new-borns. It contains propylene glycol as a solvent, which is generally considered safe. However, dosage can exceed safety thresholds in neonates, possibly causing lactic acidosis (Pouwels et al., 2019).

Clobazam tablets were reported to be too big and to have a bad taste if crushed, thus reducing children’s acceptability, in line with other studies findings. Moreover, there are frequently reported side effects including sedation, irritability, hypersalivation, and malaise, further reducing children’s compliance with the medication (Aksu Uzunhan and Gor, 2020).

Dosing valproic acid was reported as challenging, causing frequent dosing errors. Indeed, oral forms are almost completely bioavailable, but the rate of absorption varies between formulations (Guerrini, 2006). To overcome drug exposure fluctuations during a dosage interval, a modified release formulation has been approved for children. Gastrointestinal intolerance is a relatively common, dose-related adverse effect in the paediatric population.

### Immunomodulators and antineoplastics

Tablets of mercaptopurine and methylprednisolone, were reported to be poorly accepted by children because of poor taste and tablet size, while the oral liquid formulation of tacrolimus, prepared extemporaneously starting mainly from active ingredients and excipients (rather than compounded from existing adult formulations), generated various concerns regarding the stability of the formulation.

Non-adherence issues with mercaptopurine among children and adolescents are largely supported by the literature, regarding children with acute lymphoblastic leukaemia, children and adolescents with inflammatory bowel disease (Alsous et al., 2020; Hoppmann et al., 2021). Adherence issues with immunomodulators, including methylprednisolone tablets and tacrolimus, are frequently observed among children and adolescents, both for solid organ and haematopoietic stem cell transplants (Fredericks et al., 2008; Skeens et al., 2020). This heavily affects transplant outcomes in the paediatric population.

### Medicines for pain and palliative care

Oral liquid formulations of paracetamol (i.e., acetaminophen) and ondansetron, and tablets of acetylsalicylic acid were the top three problematic medicines used for pain and palliative care (in 12, eight and five cases). Dosing paracetamol in liquid formulation is complicated by the wide discrepancies among the different national and international guidelines that may cause dosing errors. Moreover, there is a risk of dosing errors due to measurement unit discrepancies (teaspoon vs. tablespoon to millilitres), and also due to the use of inappropriate dosing devices, or errors in reading the volume indications. Small absolute mistakes in volume may become large relative errors in the final dose. Furthermore, repeated errors may result in cumulative toxic effects. As a solution to this problem, the choice of a single concentration for paediatric liquid paracetamol and packaging standardisation were associated with a drop in dosing errors according to poison control centres (Kearns et al., 1998; Brass et al., 2018). Lastly, the survey confirmed that the active ingredient had a bad taste/aftertaste thus reducing child’s acceptability.

In half of the cases, acetylsalicylic acid was problematic in terms of stability of the reconstituted form or extemporaneously prepared formulation. Moreover, it was used off-label because of unapproved age or unapproved indication (three cases), generating safety concerns.

### Gastrointestinal medicines

Omeprazole in oral liquid, capsules and tablets were the three most problematic pharmaceutical forms used for diseases of the gastrointestinal tract (in 17,12 and eight cases). The oral liquid formulations required extemporaneous preparations, mostly starting from active ingredients and excipients, but also opening capsules and dispersing the product in a liquid vehicle. In 40% of cases, stability of the reconstituted formulation was reported as problematic. Moreover, solid oral formulations were not well accepted by children because of the capsule or tablet size. Also, caregivers found it difficult to determine the dose. Indeed, omeprazole capsules were used off-label with unapproved dosage and route of administration.

In line with our findings, the main issues noted with omeprazole oral liquid formulation in the literature include the uncertain stability of extemporaneous preparation, together with difficulties in formulation preparation and administration. A study in 2017 found that suspending the content of an omeprazole capsule with a liquid vehicle consisting of xanthan gum, sodium bicarbonate, compound hydroxybenzoate solution APF and purified water was stable for 30 days, at 2°C–8°C, but not at room temperature (Milic et al., 2017).

### Medicine acting on the respiratory tract

Preparation of salbutamol and budesonide for inhalation, and parenteral use as well as formulations of aminophylline were the top three problematic pharmaceutical forms of respiratory tract medicines (in 19, six and six cases). Children and caregivers found it difficult to use preparations for inhalation due to the complexity in using the administration devices, while parenteral preparations of aminophylline mainly posed dosing problems.

Suboptimal compliance with nebulized inhalation formulations of asthma medications in children, especially in pre-schoolers, has been largely reported in the literature (Gibson et al., 1996). Delivery devices play a major role in compliance to therapy and training patients in the use of these devices is key (O’Callaghan et al., 2002). Still because of the children’s immature cognitive and motor skills, devices need to be further adapted. One example is the introduction of spacers that allow medication to be administered without the need for controlling breath. However, a negative side is that the dispersion of droplets in the spray reduces the active ingredient actually administered (Cates et al., 2013; Andrzejowski and Carroll, 2016).

Intravenous aminophylline has long been known to pose dosing and safety issues mainly related to its narrow therapeutic index, ranging from 5 to 15 mcg/mL, and the need for dosing adjustment (Tuchinda et al., 1984; Kubo et al., 1986). Indeed serum theophylline levels should be checked every 12 h while the infusion is running, and continuous electrocardiogram monitoring is needed (Brit-thoracic, 2022).

### Medicines affecting the blood

Hydroxyurea capsules, oral liquid ferrous sulphate anhydrous and parenteral enoxaparin were the top three problematic pharmaceutical forms of medicines affecting the blood (in ten, six and four cases). Hydroxyurea in capsules was described as difficult to dose because of the wide range of doses across age or weight bands, discrepancies between dosing guidelines and lack of safety data. Indeed, optimal treatment with hydroxyurea requires careful attention to each patient’s treatment response (Heeney and Ware, 2010). Moreover, capsule formulations of hydroxyurea and the limited availability of the liquid formulation is an issue with acceptability for paediatric patients. Indeed, one-fourth of surveyed parents of children aged 5-17 viewed swallowing as challenging to daily medication use (Bekele et al., 2014). The extemporaneous preparation of hydroxyurea liquid formulations starting either from active ingredients and excipients, or adult formulations, was reported as a solution in 40% of cases.

Oral liquid forms of ferrous sulphate were reported to have a low child’s acceptability (70% of cases) because of an unpleasant taste/aftertaste, as well as the need for numerous daily administrations, mainly to avoid unpleasant side effects at higher dosages (BCGuidelines, 2019). Recently, an open label trial in children aged from six to 17 months found that a 2 mg/kg daily dose of a new oral ferrous sulphate heptahydrate solution provided substantial therapeutic benefit with improved tolerability in young children and caregivers (Pachuta Węgier et al., 2020).

The need for extemporaneous preparation for the paediatric parenteral form of enoxaparin was reported in half of the cases, together with related concerns about the stability of the preparation.

Indeed, dosing adaptation is needed, especially for overweight or obese children (Derbalah et al., 2022) as well as for infants and young children who require higher enoxaparin doses than adults to achieve therapeutic anti-factor Xa levels (Wysocki et al., 2021).

### Other medications

Other problematic pharmaceutical forms worth mentioning are prednisolone tablets (15 cases), parenteral preparation of amphotericin b (12 cases) and sildenafil tablets (nine cases).

Prednisolone tablets are poorly accepted by children because of an unpleasant taste/after taste, a large tablet size and the need for numerous daily administrations. Moreover, the need to reconstitute the paediatric form was noted. Such issues are once again confirmed in published data. Dose inaccuracy is a direct consequence of tablet-splitting (Haslund-Krog et al., 2019).

The parenteral preparation of amphotericin B was found difficult to dose because of the lack of pharmacokinetic evidence for the different dosing regimens and the requirement for therapeutic drug monitoring. The main issue with amphotericin B formulation is nephrotoxicity; reported effects are a decreased glomerular filtration rate and distal tubulopathy with urinary loss of potassium and magnesium, loss of urine concentrating ability, renal tubular acidosis and even Fanconi’s syndrome (Kuyucu, 2011).

Finally, sildenafil tablets were described as difficult to use because of tablets size and difficulties related to the determination of the dose given its off-label use for dose, age group and indication. Indeed, the paediatric forms had to be prepared extemporaneously, starting from the adult formulation. Stability was studied in two different suspensions and less than 2% of sildenafil citrate was degraded in the samples stored refrigerated or at room temperature in the 91-day study period, with no changes in pH, odour, or physical appearance (Nahata et al., 2006; Nahata, 2016).

### Limitations

We recognise that our study has several limitations. First, due to the nature of the survey distribution we were not able to define the response rate. Second, we did not include healthcare workers which are mainly working in the private sector such as dentists because we decided to focus on healthcare workers mainly working in the public healthcare setting. Third, the different parts of the questionnaire were arranged based on the specialization of the healthcare workers, with specific questions for some specialists. We used this approach after the evaluation of the pilot survey we conducted from December 2020 and January 2021 and the interviews with 11 respondents gathering feedback on the survey. Indeed, it was reported, for example, that pharmacists were not comfortable in answering the safety and dosing questions, nor physicians were comfortable in answering the question regarding extemporaneous preparations. Fourth, the survey was validated only in the English language. If opening the survey in Google Chrome, respondents were given the possibility use the automatic function of Google translator to translate the survey. We could not track the proportion of respondents who used this feature. Sixth, we acknowledge that our study may also have limitations in terms of the geographic distribution of respondents. Indeed, the availability and marketing of paediatric pharmaceutical dosage forms can vary significantly across different regions and countries. Still, we believe that most of the evidence gathered on the problematic issues related to paediatric pharmaceutical forms can be transposed to the underrepresented settings.

## Conclusion

To provide guidance and support for the upcoming (in 2023) revision of the EMLc, the opinion of experts and the experience of front-line healthcare providers was gathered.

Our online survey investigated multiple aspects of medicines for children: acceptability from a child’s perspective, ease of use by caregivers, need for extemporaneous preparation, off-label use, concerns regarding pharmacokinetics, dose selection and safety, need for accelerated paediatric development, lack of child-appropriate formulations and obstacles to access. The responses provided reflect the perspective of healthcare workers around the world, particularly in the European region, in the African region, and in the Region of the Americas, with limited representation from Northern Africa and the Middle East.

Due to the diversity of national healthcare systems, access to care varies from country to country and medical costs are often not covered or reimbursed, especially in low-middle income countries. Together with the general scarcity of marketed formulations for children, this challenges the fulfilment of the universal right to healthcare, especially for children.

Indeed, before specific products can be considered for inclusion in the EMLc or enter the GAP-f prioritization and acceleration process, these results need to be analysed in conjunction with the ongoing work of others involved in the upcoming revision of the EMLc, namely, an expert review of the products already available in the EMLc and a detailed analysis of the global market of paediatric medicines. As previously reported, efforts to develop oral formulations for vulnerable populations, such as paediatric patients, should be accelerated so that the uncertainties associated with off-label drug preparation and use are minimised and therapeutic benefits are optimised (Singer and Zaïr, 2016; Parrish et al., 2022).

## Data Availability

The raw data supporting the conclusion of this article will be made available by the authors, without undue reservation.
