# Supplementary material for: Paediatric medicine issues and gaps from healthcare workers point of view: survey results and a narrative review from the global accelerator for paediatric formulations project
Source: Front Pharmacol. 2023 Jul 17;14:1200848. doi: 10.3389/fphar.2023.1200848 (PMC10390094; doi:10.3389/fphar.2023.1200848)
Supplement: Supplementary file 4 [file DataSheet1.docx]

**Paediatric medicine issues and gaps from healthcare workers point of view: survey results and a narrative review from the Global Accelerator for Paediatric formulations project**

Elisa Barbieri, PharmD ^1,2^, Chiara Minotti, MD ^1^, Sara Cavagnis MD^2^, Carlo Giaquinto MD^1,2^, Bernadette Cappello MPH^3^, Martina Penazzato MD^4^, Marc Lallemant MPH^2,5^

**Affiliations:**

^1^ Division of Pediatric Infectious Diseases, Department of Women’s and Children’s Health, University of Padova, 35100 Padova, Italy

^2^ Penta – Child Health Research, 35100 Padova, Italy

^3^ Department of Health Products Policy and Standards, World Health Organization, 1211 Geneva, Switzerland

^4^ WHO Research for Health Department, World Health Organization, 1211 Geneva, Switzerland

^5^ Faculty of Associated Medical Sciences, Chiang Mai University, Chiang Mai, Thailand

**Corresponding Author:** Elisa Barbieri

via Giustiniani 3, 35100 Padova (PD), Italy

E-mail: elisa.barbieri@unipd.it

Tel: +39 049 964 0122, Fax: +39 049 964 0123

Table of Contents

[REDcap online survey template 2](#_Toc131600443)

[List of physicians societies contacted 7](#_Toc131600444)

[List of family medicine physician societies contacted 9](#_Toc131600445)

[List of nurses societies contacted 10](#_Toc131600446)

[List of pharmacists/pharmaceutical societies contacted 12](#_Toc131600447)

[Figure S1. Flow chart of survey respondents’ inclusion and exclusion criteria 14](#_Toc131600448)

[Figure S2. Map of the countries where the respondents practice weighted by the number of medicines mentioned in the survey (panel A) and map of the countries where physicians (panel B), nurses (panel C) and pharmacists (panel D) responding to the survey practice. 15](#_Toc131600449)

[Table S1. Distribution of the sub-specialties and the therapeutic classes of the medicines of the physicians, nurses, and pharmacists reporting medicines as problematic. 16](#_Toc131600450)

[Table S2. Missing products for paediatric care according to physicians, nurses, and pharmacists. 18](#_Toc131600451)

[Table S3. Missing products for paediatric care according to physicians, nurses, and pharmacists by pharmaceutical form. 18](#_Toc131600452)

[Table S4. Class of products considered problematic. 18](#_Toc131600453)

[Table S5. Products considered most problematic for physicians, nurses, and pharmacists, stratified by pharmaceutical form [Legend: (*) Physicians, (**) Pharmacists, (^) Physicians and nurses, (^^) Physicians and pharmacists.] 19](#_Toc131600454)

### REDcap online survey template

**Paediatric formulation gap survey**

We thank you for agreeing to participate in our survey!

Our first objective is to collect your personal experience with medicines for children in your field of expertise, identifying gaps in the paediatric formulations that you think are the most important.

You can save the survey and come back to it at any time.

If you would like to know more about the WHO **Global Accelerator for Paediatric Formulation (GAP-f)**, please click [here](http://gap-f.org/).

**Before starting the survey, we need your consent to process your data in compliance with Regulation (EU) 2016/679 General Data Protection Regulation (GDPR) and the Legislative Decree n. 196 of 30 June 2003 "Personal data protection Code" (please read the Privacy Notice). Do you agree?**

(Please note, if you do not give your consent, you will not be able to take part in the survey.)

- Yes
- No

**First Name**

__________________________________

**Last Name**

__________________________________

**Email address**

__________________________________

**Country where you are working** (Drop-down list)

**Please indicate if you are collaborating or conducting work/research in another region/country**

__________________________________

**Do you work mainly in the hospital or the primary care setting?**

- Hospital
- Primary care

**Please indicate your specialty**

- Pharmacist
- Medical Doctor
- Nurse

**Please indicate your sub-specialty or specify the clinical fields in which you practice**

- Allergy and immunology
- Cardiology
- Dermatology
- Ear-Nose-Throat specialist
- Emergency medicine
- Endocrinology
- Gastroenterology
- General paediatrics
- Infectious diseases
- Intensive care
- Neonatology
- Nephrology
- Neurology
- Nutrition
- Onco-haematology
- Psychiatry
- Pulmonology
- Surgery
- Other ______

**In your practice, what are the medicine classes you prescribe/deal with the most?**

Please tick all that apply from the list.

For your information, we have listed the medicines classes as per WHO EMLc

**Please select at least three paediatric formulations that you find problematic and provide the**

**reasons. [ONLY FOR MEDICAL DOCTORS, repeated 7 times (3 compulsory and 4 optional]**

1. **Medicine** (drop down list with general name)

**Formulation:**

- Tablet
- Capsule
- Oral liquid
- Parenteral preparations
- Rectal preparations
- Preparation for inhalation
- Topical
- Other _____________

**Main reasons** – please tick all that apply:

- **Poor acceptability** – children find it difficult to take
  - Taste/aftertaste
  - Tablet/capsule size
  - High volume of liquid for a single dose
  - Numerous daily administrations
  - Texture/appearance/smell
  - Complexities in using the device (i.e., respiratory device, insulin pump)
  - Other – please specify ______
- **Difficulties in handling by parents**
  - Complex preparation (i.e., problematic suspension reconstitution)
  - Determination of the dose
  - Need for a vehicle (i.e., soft food or liquid/water)
  - Problematic administration device (i.e., respiratory device)
  - Problematic packaging opening/closing
  - Need to be stored at a specific temperature
  - Other _______
- **Off-label use**
  - Unapproved indication
  - Unapproved age group
  - Unapproved dosage
  - Unapproved route of administration
- **Dosing and safety issues**
  - Discrepancies between dosing guidelines
  - Very wide range of dose across age/weight bands
  - Lack of PK evidence for dosing regimen
  - Lack of safety data
  - Narrow therapeutic index
  - Requirement for therapeutic drug monitoring
  - Frequent dosing errors (i.e., the formulation is too concentrated)
  - Other _______
- **Use in special situations** (such as malnourished patients or children with comorbidities)
  - Lack of indication for specific use
  - Lack of safety data
  - Lack of specific dosing guidelines
  - Drug-drug interaction
  - Lack of PK data
  - Medication burden (the child is taking too many medicines daily)
  - Other _________

**Other specifications:** if you selected off-label use or special situations as issues, please provide further explanation

**Please select at least three paediatric formulations that you find problematic and provide the**

**reasons. [ONLY FOR PHARMACISTS, repeated 7 times (3 compulsory and 4 optional]**

1. **Medicine** (drop down list)

**Formulation:**

- Tablet
- Capsule
- Oral liquid
- Parenteral preparations
- Rectal preparations
- Preparations for inhalation
- Topical
- Other _____________

**Main reasons** – please tick all that apply:

- **Requirement for extemporaneous preparation:** The formulation is prepared
  - Starting from the active ingredients and excipients
  - Modifying the adult formulation
- **Concerns regarding reconstitution and stability**
  - Access to clean water for formulation reconstitution
  - Unclear or too complex label instructions
  - Numerous steps required for reconstitution
  - Need for dilution to enable the correct dose to be accurately measured
  - Problematic stability (i.e. flocculation, rapid deterioration/oxidation)
  - Other – please specify _______
- **Off-label use**
  - Unapproved indication
  - Unapproved age group
  - Unapproved dosage
  - Unapproved route of administration
- **Use in special situations** (such as malnourished patients or children with comorbidities)
  - Lack of indication for specific useLack of safety data
  - Lack of specific dosing guidelines
  - Drug-drug interaction
  - Lack of PK data
  - Medication burden (the child is taking too many medicines daily)
  - Other _________

**Other specifications:** if you selected off-label use or special situations as issues, please provide further explanation

**Please name at least three paediatric formulations that you find problematic and provide the**

**reasons. [ONLY FOR NURSES, repeated 7 times (3 compulsory and 4 optional]**

1. **Medicine** (drop down list)

**Formulation:**

- Tablet
- Capsule
- Oral liquid
- Parenteral preparations
- Rectal preparations
- Preparations for inhalation
- Topical
- Other _____________

**Main reasons** – please tick all that apply:

- **Poor acceptability** – children find it difficult to take
  - Taste/aftertaste
  - Tablet/capsule size
  - High volume of liquid for a single dose
  - Numerous daily administrations
  - Texture/appearance/smell
  - Complexities in using the device (i.e., respiratory device, insulin pump)
  - Other ______
- **Usability issues**
  - Complex preparation (i.e., problematic suspension reconstitution)
  - Need for dilution to enable the correct dose to be accurately measured
  - Determination of the dose
  - Need for a vehicle (i.e., soft food or liquid/water)
  - Problematic administration device (i.e., respiratory device)
  - Problematic packaging opening/closing
  - Need to be stored at a specified temperature
  - Other – please specify_______
- **Other issues - please specify**________

**Missing formulations - What are the paediatric formulations that are clearly missing? [FOR EVERYONE, repeated 5 times]**

Please give three examples and indicate the reason(s).

1. **Medicine (drop down list)**

**Formulation:**

- Tablet
- Capsule
- Oral liquid
- Parenteral preparations
- Rectal preparations
- Preparations for inhalation
- Topical
- Other _____________

**Formulation strength (if relevant)**______________

**Reasons** – please tick all that apply

- Only available as adult formulation (i.e. tablet, capsules)
- Not licensed in my country
- Only available in the private sector
- Overly expensive
- Not available in my setting because it’s not not in institution’s drug formulary Not available in my setting because of frequent shortages
- Other – please specify _________

**If you have any other comments on missing formulations or related issues, please write them here**

**Can we contact you to further discuss some of the shortcomings you have noted?**

- Yes
- No

### List of physicians societies contacted

| **Society name** | **Country/area** |
| --- | --- |
| Afghan Pediatric Association | Afghanistan |
| Egyptian Pediatric Society | Egypt |
| Iranian Society Of Pediatrics | Iran |
| Jordan Pediatric Society | Jordan |
| Lebanese Pediatric Society | Lebanon |
| Pediatric Society Palestine | Palestine |
| Sudan Association of Paediatricians | Sudan |
| Italian-Arabic Pediatric Society | Italy-Arab countries |
| Societe Beninoise de Pediatrie | Benin |
| Pediatric Society of Burkina Faso | Burkina Faso |
| Cambodian Pediatric Association | Cambodia |
| Societe Camerounaise de Pediatrie | Cameroon |
| Societe Congolaise de Pediatrie | Congo |
| Society Pediatric Congo Democratique Republic | DRC |
| Association des Pédiatres d'Afrique Noire Francophone | Francophone Africa |
| Societe Haitienne de Pediatrie | Haiti |
| Societe de Pediatrie du Laos | Laos |
| Association Nigerienne de Pediatrie | Niger |
| Societe Senegalaise de Pediatrie | Senegal |
| Societe Togolaise de Pediatrie | Togo |
| Vietnam Pediatric Association | Vietnam |
| Pediatres du Monde | Global |
| Societe Algerienne de Pediatrie | Algeria |
| Libyan Pediatric Society | Libya |
| Societe Marocaine de Pediatrie | Morocco |
| Societe Tunisienne de Pediatrie | Tunisia |
| Sociedade Angolana de Pediatria | Angola |
| Sociedad Brasilera de Pediatra | Brasil |
| Sociedad Argentina de Pediatra | Argentina |
| Sociedad Boliviana de Pediatria | Bolivia |
| Sociedad Colombiana de Pediatra | Colombia |
| Asociacin Costarricense de Pediatra | Costa Rica |
| Sociedad Cubana de Pediatra | Cuba |
| Sociedad Ecuatoriana de Pediatra | Ecuador |
| Asociacion de Pediatria de El Salvador | El Salvador |
| Asociacion Pediatrica de Guatemala | Guatemala |
| Honduran Pediatric Association | Honduras |
| Asociacion Mexicana de Pediatria | Mexico |
| Confederacion Nacional de Pediatria de Mexico | Mexico |
| Sociedad Nicaragense de Pediatra | Nicaragua |
| Sociedad Paraguaya de Pediatria | Paraguay |
| Sociedade Peruana de Pediatria | Peru |
| Philippine Pediatric Society | Philippines |
| Sociedad Dominicana de Pediatra | Republica Dominicana |
| Sociedad Venezolana de Puericultura y Pediatria | Venezuela |
| Sociedad Latino Americana de Cuidados Intensivos Pediatricos | Latin America |
| Asociación Latinoamericana de Nefrología Pediátrica (ALANEPE) | Latin America |
| Latin American Thorax association (ALAT) | Latin America |
| Sociedad Latinoamericana de Emergencias Pediátricas (SLEPE) | Latin America |
| Red de Investigacion y Desarollo de la Emergencia Pediatrica Latinoamerica (RIDEPLA) | Latin America |
| Latinamerican Society for Pediatric Gastroenterology, Hepatology and Nutrition (LASPGHAN) | Latin America |
| Paediatric Association of Jamaica | Jamaica |
| PATS pediatric working group | Africa |
| Kenya Paediatric Association | Kenya |
| Latin American Pediatric Association | Latin America |
| Malaysian Paediatric Association | Malaysia |
| Myanmar Pediatric Society | Myanmar |
| Paediatric Association of Nigeria | Nigeria |
| Zambia Paediatric Association | Zambia |
| Sociedad Mexicana de Neurologia Pediatrica | Mexico |
| International Association for Child and Adolescent Psychiatry and Allied Professions | International |
| International Society for Pediatric and Adolescent Diabetes (ISPAD) | Global |
| Commonwealth Association of Paediatrics Gastroenterology and Nutrition (CAPGAN) | Commonwealth |
| Asia Pacific Association of Allergy, Asthma and Clinical Immunology (APAAACI) | Asia |
| Paediatric Cardiac Society of South Africa | South Africa |
| Pediatric Emergency Research Networks (PERN) | Global |
| ASPAE (African Society for Pediatric and Adolescents Endocrinology) | Africa |
| Asian Pan-Pacific Society for Pediatric Gastroenterology, Hepatology and Nutrition (APPSPGHAN) | Asia |
| Pan-Arab Society for Paediatric Gastroenterology, Hepatology and Nutrition (PASPGHAN) | Panarab |
| Federation of International Societies of Pediatric Gastroenterology, Hepatology and Nutrition (FISPGHAN) | Global |
| Albanian Pediatric Society | Albania |
| Armenian Pediatric Association | Armenia |
| APPA (Asia Pacific Pediatric Association) | Asia |
| Bangladesh Pediatric Association | Bangladesh |
| Botswana Pediatric Association | Botswana |
| Chinese Pediatric Society, Chinese Medical Association | China |
| Ethiopian Pediatric Society | Ethiopia |
| Paediatric Society of Ghana | Ghana |
| The Indian Academy of Pediatrics | India |
| Indonesian Pediatric Society | Indonesia |
| Pediatric Society of Republic Kosovo | Kosovo |
| Malawi Pediatric Association | Malawi |
| Mongolian Academy of Pediatrics | Mongolia |
| Association of preventive pediatrics of Montenegro | Montenegro |
| Nepal Paediatric Society | Nepal |
| Pakistan Paediatric Association | Pakistan |
| Union of Paediatricians of Russia | Russia |
| Paediatric Association of Serbia | Serbia |
| South African Paediatric Association | South Africa |
| Sri Lanka College of Paediatricians | Sri Lanka |
| Association of Pediatric Surgeons, Anesthesiologists, and Reanimatologists the Republic of Tajikistan | Tajikistan |
| Paediatric Association of Tanzania | Tanzania |
| Pediatric Society of Thailand | Thailand |
| Paediatric Association of The Gambia | The Gambia |
| Turkish National Pediatric Society | Turkey |
| Turkish Pediatric Association | Turkey |
| Uganda Paediatric Association | Uganda |
| Association of Ukrainian Pediatricians | Ukraine |
| Uzbekistan Pediatric Association | Uzbekistan |
| Paediatric Association of Zimbabwe | Zimbabwe |
| African Paediatric Nephrology Association (AFPNA) | Africa |
| Asian Pediatric Nephrology Association (AsPNA) | Asia |
| International Pediatric Nephrology Association (IPNA) | Global |
| Turkish Child Neurology Association | Turkey |
| Pediatric and Child Health Association (PACHA) | Malawi |
| International Society of Tropical Pediatrics | International |
| UNICEF - Copenaghen | Global |
| Foundation for Advancement of International Medical Education and Research | Global |
| International Society of Pediatric Dermatology | Global |
| World Federation of Associations of Pediatric Surgeons | Global |
| National Neonatology Forum of India | India |
| United South African Neonatal Association | Sounth Africa |
| Sociedad IBEROAMERICANA de Neonatología | Latin America |
| VIETNAM INSTITUTE OF APPLIED MEDICINE | Vietnam |
| Perinatal Society of Cambodia | Cambodia |
| Angkor Hospital for Children | Cambodia |

### List of family medicine physician societies contacted

| **Society name** | **Country** |
| --- | --- |
| Chinese Society of General Practice | China |
| Cross-Straits Medicine Exchange Association Committee of International and Premium Medicine | China |
| Fiji College of General Practitioners | Fiji |
| Indonesian Association of Family Physicians | Indonesia |
| Indonesian Society of Teachers in Family Medicine | Indonesia |
| Japan Primary Care Association | Japan |
| Korean Academy of Family Medicine | Korea |
| Academy of Family Physicians of Malaysia | Malaysia |
| Family Medicine Specialist Association of Malaysia | Malaysia |
| Mongolian Association of Family Medicine Specialists | Mongolia |
| Philippine Academy of Family Physicians | Philippines |
| College of Family Physicians Singapore | Singapore |
| Chinese Taipei Association of Family Medicine | Taiwan |
| Royal Australian College of General Practitioners | Australia |
| Royal New Zealand College of General Practitioners | New Zealand |
| The General Practitioners/Family Physicians Association of Thailand | Thailand |
| The Hong Kong College of Family Physicians | Hong Kong |
| Academy of Family Physicians of India | India |
| Bangladesh College of General Practitioners | Bangladesh |
| College of Family Medicine Pakistan | Pakistan |
| College of General Practitioners of Sri Lanka | Sri Lanka |
| Federation of Family Physicians' Associations of India | India |
| Indian Medical Association College of General Practitioners | India |
| Pakistan Society of Family Physicians, Lahore | Pakistan |
| Association of General and Private Medical Practitioners of Nigeria | Nigeria |
| College of Primary Care Physicians of Zimbabwe | Zimbabwe |
| Kenya Association of Family Physicians | Kenya |
| Medcamer Family Medicine | Cameroon |
| National Postgraduate Medical College of Nigeria, Faculty of Medicine | Nigeria |
| South African Academy of Family Physicians | South Africa |
| Rural Doctors Association of Southern Africa | South Africa |
| The Society of Family Physicians of Ghana | Ghana |
| WONCA Africa Region | Global |
| Wonca Asia Pacific region | Asia |
| Association of General Practitioners of Russian Federation | Russia |
| Armenian Association of Family Physicians | Armenia |
| *Association of Doctors of General Practice/Family Medicine of North Macedonia* | North Macedonia |
| Association of Family Physicians of Kosovo | Kosovo |
| Association of Family Physicians of Republic of Srpska | Bosnia and Herzegovina |
| Association of Family Physicians of the Federation of Bosnia & Herzegovina | Bosnia and Herzegovina |
| *Bulgarian General Practice Society for Research And Education* | Bulgaria |
| Family Group Practice and Nurses Association of Kyrgyzstan | Kyrgzstan |
| Georgia Family Medicine Association | Georgia |
| Israel Association of Family Physicians | Israel |
| Kazakhstan Association of Family Physicians | Kazakhstan |
| Public Organization National Association of Family Medicine Staff of Tajikistan | Tajikistan |
| Serbian Medical Association / Section of General Practice | Serbia |
| Turkish Association of Family Physicians | Turkey |
| Ukrainian Family Medicine Association | Ukraine |
| *Caribbean College of Family Physicians* | Caribbean |
| Brazilian Society of Family and Community Medicine | Brasil |
| Federacion Argentina de medicina familiar y general | Argentina |
| Chilean Society of Family Medicine | Chile |
| Colombian Society of Family Medicine | Colombia |
| Sociedad Cubana de Medicina Familiar | Cuba |
| Sociedad Boliviana de Medicina Familiar | Bolivia |
| Costa Rican Association of Specialists in Family and Community Medicine | Costa Rica |
| Sociedad Dominicana de Medicina Familiar y Comunitaria | Dominican Republic |
| Sociedad Ecuatoriana de Medicina Familiar | Ecuador |
| Mexican Federation of Family Medicine Specialists and Residents | Mexico |
| Nicaraguan Association of Family Medicine | Nicaragua |
| Panamanian Association of Family Medicine | Panama |
| Paraguayan Society of Family Medicine | Paraguay |
| Peruvian Society of Family and Community Medicine | Peru |
| Salvadorean Family Physicians Association | El Salvador |
| Uruguayan Society of Family ​and Community Medicine | uruguay |

### List of nurses societies contacted

| **Society name** | **Country** |
| --- | --- |
| Bahrain Nursing Society | Bahrain |
| Egyptian Nurses Syndicate | Egypt |
| Iranian Nursing Organization | Iran |
| Order of Nurses in Lebanon | Lebanon |
| Oman Nursing Association | Oman |
| Palestinian Nursing and Midwifery Association | Palestine |
| Saudi NursesAssociation | Saudi Arabia |
| Emirates Nursing Association | United Emirates |
| Association Professionnelle des Infirmiers/ères du Burkina | Burkina Faso |
| Ordre National des Infirmiers de la RDC | DRC |
| Association Nationale Francaise des Infirmieres et Infirmiers Diplomes et des Etudiants (ANFIIDE) | France |
| Association Nationale des Infirmières et Infirmiers d'Etat du Sénégal | Senegal |
| Rwanda Nurses and Midwives Union (RNMU) | Rwanda |
| Nurses Association of the Republic of Seychelles | Seychelles |
| Association Marocaine des Sciences Infirmières et Techniques Sanitaires | Morocco |
| Nursing Council Of Angola (ORDENFA) | Angola |
| Ordem dos enfermeiros | Angola |
| Conselho Federal de Enfermagem | Brasil |
| Federación Argentina de Enfermería | Argentina |
| Colegio de Enfermeras de Chile | Chile |
| Asociación Nacional de Enfermeras de Colombia | Colombia |
| Colegio de Enfermeras de Costa Rica | Costa Rica |
| Sociedad Cubana de Enfermería | Cuba |
| Colegio Dominicano de Profesionales de Enfermería | Dominican Republic |
| Federación Ecuatoriana de Enfermeras/os | Ecuador |
| Asociación Nacional de Enfermeras de El Salvador | El Salvador |
| Asociación Guatemalteca de Enfermeras Profesionales | Guatemala |
| Colegio de Profesionales de Enfermería de Honduras | Honduras |
| Colegio Nacional de Enfermeras | Mexico |
| Federación Mexicana de Colegios de Enfermería | Mexico |
| Asociación de Enfermeras Nicaragüenses | Nicaragua |
| Asociación Nacional de Enfermeras de Panamá | Panama |
| Asociación Paraguaya de Enfermeras | Paraguay |
| Colegio de Enfermeros del Perú | Perù |
| Philippine Nurses Association | Philippines |
| Colegio de Enfermeras del Uruguay | Uruguay |
| Nursing and Midwifery council of Nigeria | Nigeria |
| Myanmar Nurse and Midwife Association | Myanmar |
| Association Suisse des Infirmières/Infirmiers | Switzerland |
| Malaysian Nurses Association | Malaysia |
| Sierra Leone Nurses Association | Sierra Leone |
| Nurses and Midwives Association of Slovenia | Slovenia |
| Sri Lanka Nurses Association | Sri Lanka |
| Association nationale des infirmiers/ères du Togo | Togo |
| Uganda Nurses & Midwives Union | Uganda |
| Zambia Union of Nurses Organization | Zambia |
| Turkish Nurses Association | Turkey |
| Tanzania National Nurses' Association | Tanzania |
| Lithuanian Nurses Association | Lithuania |
| American Nurses Association | USA |
| Australian College of Nursing | Australia |
| Colegio de Enfermeras de Bolivia | Bolivia |
| Norwegian Nurses Organisation | Norway |
| East Timor Nurses Association | East Timor |
| Collegi d'Infermeres i Llevadores d'Andorra | Andorra |
| Österreichischer Gesundheits -und. Krankenpflegeverband | Austria |
| Nurses Association of the Commonwealth of the Bahamas | Bahamas |
| Bangladesh Nurses Association | Bangladesh |
| Barbados Nurses Association | Barbados |
| Fédération Nationale des Infirmières de Belgique (FNIB) | Belgium |
| Nurses Association of Belize | Belize |
| Bermuda Nurses' Association | Bermuda |
| Botswana Nurses Union | Botswana |
| Bulgarian Association of Health Professionals in Nursing | Bulgaria |
| Canadian Nurses Association | canada |
| Chinese Nursing Association | China |
| Cook Islands Nurses Association | Cook Islands |
| Croatian Nurses Association | Croatia |
| Cyprus Nurses and Midwives Association | Cyprus |
| Danish Nurses' Organization | Denmark |
| Eritrean Nurses Association | Eritrea |
| Estonian Nurses Union | Estonia |
| Swaziland Nursing Association | Swaziland |
| Ethiopian Nurses Association | Ethiopia |
| Finnish Nurses Association | Finland |
| National Association of Gambia Nurses & Midwives | Gambia |
| DBfK -Bundesverband | Germany |
| Ghana Registered Nurses and Midwives Association | Ghana |
| Ghana college of nurses and midwives | Ghana |
| Hellenic National Nurses Association | Greece |
| Grenada Nurses Association | Grenada |
| Guyana Nurses Association | Guyana |
| College of Nursing Hong Kong | Hong Kong |
| Icelandic Nurses Association | Iceland |
| Indian Nursing Council | India |
| Persatuan Parawat Nasional Indonesia | Indonesia |
| Irish Nurses and Midwives Organisation | Ireland |
| National Association of Nurses in Israel | Israel |
| Consociazione Nazionale delle Associazioni Infermiere-Infermieri | Italy |
| Nurses Association of Jamaica | Jamaica |
| Japanese Nursing Association | Japan |
| Jordan Nurses and Midwives Council | Jordan |
| National Nurses association of Kenya | Kenya |
| Korean Nurses Association | Korea |
| Kuwait Nursing Association | Kuwait |
| Lesotho Nurses Association | Lesotho |
| Liberia Nurses Association | Liberia |
| Association Nationale des Infirmiers & Infirmières du Luxembourg | Lusembourg |
| Nurses Association of Macau | Macau |
| National Organisation of Nurses and Midwives of Malawi | Malawi |
| Malta Union of Midwives & Nurses | Malta |
| Mauritius Nursing Association | Mauritius |
| Association nationale des infirmières de Monaco | Monaco |
| Mongolian Nurses Association | Mongolia |
| National Association of Nurses and Midwives of Montenegro | Montenegro |
| Namibian Nursing Association | Namibia |
| Nursing Association of Nepal | Nepal |
| Nieuwe Unie’91 -NU' 91 | Netherlands |
| New Zealand Nurses' Organisation | New Zealand |
| North Macedonian Nurses and Midwives Association | North Macedonia |
| Pakistan Nurses Federation | Pakistan |
| Polskie Towarzystwo Pielegniarskie | Poland |
| Ordem dos Enfermeiros | Portugal |
| Romanian Nursing Association | Romania |
| The Order of Nurses, Midwives and Medical Assistants in Romania | Romania |
| Russian Nurses Association | Russia |
| Samoa Registered Nurses Association | Samoa |
| Associação Nacional dos Enfermeiros y parteiros de São Tomé e Príncipe | Sao Tomé e Principe |
| Association of Health Workers of Serbia | Serbia |
| Singapore Nurses' Association | Singapore |
| Solomon Islands Nurses Association | Solomon Islands |
| Somaliland Nursing and Midwifery Association | Somaliland |
| Democratic Nursing Organization of South Africa | South Africa |
| South Sudan Nurses and Midwives Association | South Sudan |
| Consejo General de Enfermería de España | Spain |
| St. Lucia Nurses Association | St Lucia |
| Suriname Nurses Association | Suriname |
| Joint Virtual Swedish Nurse Organisation-for international work | Sweden |
| Taiwan Nurses Association | Taiwan |
| Nurses Association of Thailand | Thailand |
| Tonga Nurses Association | Tonga |
| Trinidad & Tobago Registered Nurses Association | Trinidad and Tobago |
| Zimbabwe Nurses Association | Zimbabwe |
|  |  |
| Asia Pacific Paediatric Nurses Association | Asia |
| World Federation of Critical Care Nurses | Global |
| Council of International Neonatal Nurses (COINN) | Global |
| North American Nursing Diagnosis Association (NANDA) | Global |
| International Family Nursing association | Global |
| International Council of Nursing | Global |

### List of pharmacists/pharmaceutical societies contacted

| Society name | Country |
| --- | --- |
| Emirates Medical Association: Emirates Pharmacy Society | Arab emirates |
| Lebanese order of Pharmacists | Lebanon |
| Syndicate of Iraqi Pharmacists | Iraq |
| Jordan Pharmacists Association | Jordan |
| Conseil National de l'Ordre des Pharmaciens de Côte d'Ivoire | Côte d'Ivoire |
| Conseil National de l'Ordre des Pharmaciens de la République Démocratique du Con | DRG |
| Conseil National de l'Ordre des Pharmaciens du mali | Mali |
| National Council of Pharmacists in Cameroon | Cameroon |
| Ordre National des Pharmaciens de Madagascar | Madagascar |
| Ordre national des Pharmaciens de la République du Congo | Congo |
| Ordre National des Pharmaciens du Burkina Faso | Burkina Faso |
| Ordre National des Pharmaciens du Tchad | Tchad |
| Pharmaceutical Association of Mauritius | Mauritius |
| Pharmaceutical Society of Sierra Leone | Sierra Leone |
| Syndicat National des Pharmaciens Praticiens Hospitaliers et Universitaires | France |
| Vietnam Young Pharmacists Association | Vietnam |
| Conseil National de l'Ordre des Pharmaciens d'Algérie, | Algerie |
| Conseil national de l'Ordre des pharmaciens du Burundi | Burundi |
| Ordre des pharmaciens du Cambodge | Cambodia |
| Ordre national des pharmaciens, médecins, chirurgiens-dentistes et biologistes médicaux des Comores | Comores |
| Ordre national des pharmaciens du Gabon | Gabon |
| Conseil national de l'Ordre des pharmaciens de Guinée | Guineè |
| Association des pharmaciens d'Haïti, | Haiti |
| Conseil national de l'Ordre des Médecins, Chirurgiens-dentistes et Pharmaciens Niger | Niger |
| Ordre des pharmaciens de Nouvelle-Calédonie | Nouvelle-Calédonie |
| Ordre des pharmaciens du République Centrafricaine | Central Africa Republic |
| Ordre des pharmaciens du Rwanda | Rwanda |
| Ordre des Pharmaciens du Sénégal | Senegal |
| Conseil national de l'Ordre des pharmaciens du Togo | Togo |
| Ordre national des Médecins, Pharmaciens et Chirurgiens-dentistes - Section des pharmaciens de Mauritanie | Mauritanie |
| Fédération Algérienne de Pharmacie | Algerie |
| Liberia Pharmacy Board | Liberia |
| Conseil national de l'Ordre des pharmaciens du Maroc | Maroc |
| Conseil national de l'Ordre des pharmaciens de Tunisie | Tunisie |
| Colegio de Farmacéuticos de Costa Rica | Costa Rica |
| Colegio de Químicos Farmacéuticos y Bioquímicos Farmacéuticos de Pichincha | Bolivia |
| Confederación Farmacéutica Argentina | Argentina |
| [Farmaceuticos sin Fronteras Argentina](mailto:info@fsfargentina.org.ar?Subject=Contacto%20web) | Argentina |
| Sociedad Boliviana de Ciencias Farmaceuticas | Bolivia |
| Asociación de Química y Farmacia del Uruguay, | Uruguay |
| Colegio Nacional de Farmacéuticos de Panamá, | Panama |
| Colegio Nacional de Químicos Farmacéuticos de Colombia, | Colombia |
| FEDERACION FARMACEUTICA SUDAMERICANA | Global |
| Cyprus Turkish Pharmacist Association | Turkey |
| Brazilian Federal Council of Pharmacy | Brazil |
| Community Pharmacy Owner Syndicate | Yemen |
| Philippine Pharmacists Association | Philippines |
| Rwanda National Pharmacy Council | Rwanda |
| Japan Pharmaceutical Association | Japan |
| All-Ukrainian Pharmaceutical Chamber | Ukraine |
| Afghanistan Nationwide Pharmacists Association | Afghanistan |
| Association of Community Pharmacists of Nigeria | Nigeria |
| Association of Mongolian Pharmacy Professionals | Mongolia |
| Bangladesh Pharmaceutical Society | Bangladesh |
| Academy of Pharmaceutical Sciences of Brazil, | Brazil |
| Chamber of Pharmacists of the Federation of Bosnia & Herzegovina | Bosnia & Herzegovina |
| Chinese Pharmaceutical Association | China |
| Ethica Independent Pharmacies Association | Jordan |
| Ethiopian Pharmaceutical Association | Ethiopia |
| Indian Association of Colleges of Pharmacy | India |
| Indian Pharmaceutical Association | India |
| Indonesian Pharmacist Association | Indonesia |
| Iranian Association of Pharmaceutical Scientists | Iran |
| Japanese Society of Hospital Pharmacists | Japan |
| Kosova Chamber of Pharmacists | Kosovo |
| Malaysian Pharmaceutical Society | Malasya |
| Moscow Pharmaceutical Society | Russia |
| National Association of Hospital Pharmacists of Romania | Romania |
| National College of Pharmacists | India |
| Nepal Pharmaceutical Association | Nepal |
| Pakistan Pharmacists Association | Pakistan |
| Pharmacy Graduates’ Association of Pakistan | Pakistan |
| Pharmaceutical Chamber of Macedonia | Macedonia |
| Pharmaceutical Chamber of Montenegro | Montenegro |
| Pharmaceutical Chamber of Serbia | Serbia |
| Pharmacy Council, Ghana | Ghana |
| Pharmaceutical Society of Kenya | Kenya |
| Pharmaceutical Society of Korea | Korea |
| Pharmaceutical Society of South Africa | South Africa |
| Pharmaceutical Society of Tanzania | Tanzania |
| Pharmaceutical Society of Sri Lanka | Sri Lanka |
| Pharmaceutical Society of Zambia | Zambia |
| Pharmaceutical Society of Uganda | Uganda |
| Pharmaceutical Society of Zimbabwe | Zimbabwe |
| Pharmacists Order of Albania | Albania |
| Japanese Society of Pharmaceutical Sciences | Japan |
| Pharmaceutical Society of Japan | Japan |
| Arab Association of Pharmacy Progress | Panarab |
| ReMed | Francophone countries |
| Association de Pharmacie Galénique Industrielle | Francophone countries |
| Indian pharmacist Association | India |
| Farmaceutsko društvo Republike Srpske | Bosnia |
| Pharmaceutical Society Of Kenya | Kenya |
| Türk Eczacıları Birliği | Turkey |
| FSF - Farmacien sans frontiers | Global |
| African chapter FIP | Africa |
| Western Pacific Pharmaceutical Forum | West Pacific |
| National Pharmaceutical Association of the South East Asian Region (SEAR) Pharm Forum | South Asia |
| Pharmaceutical Forum of the Americas | America |

### Figure S1. Flow chart of survey respondents’ inclusion and exclusion criteria


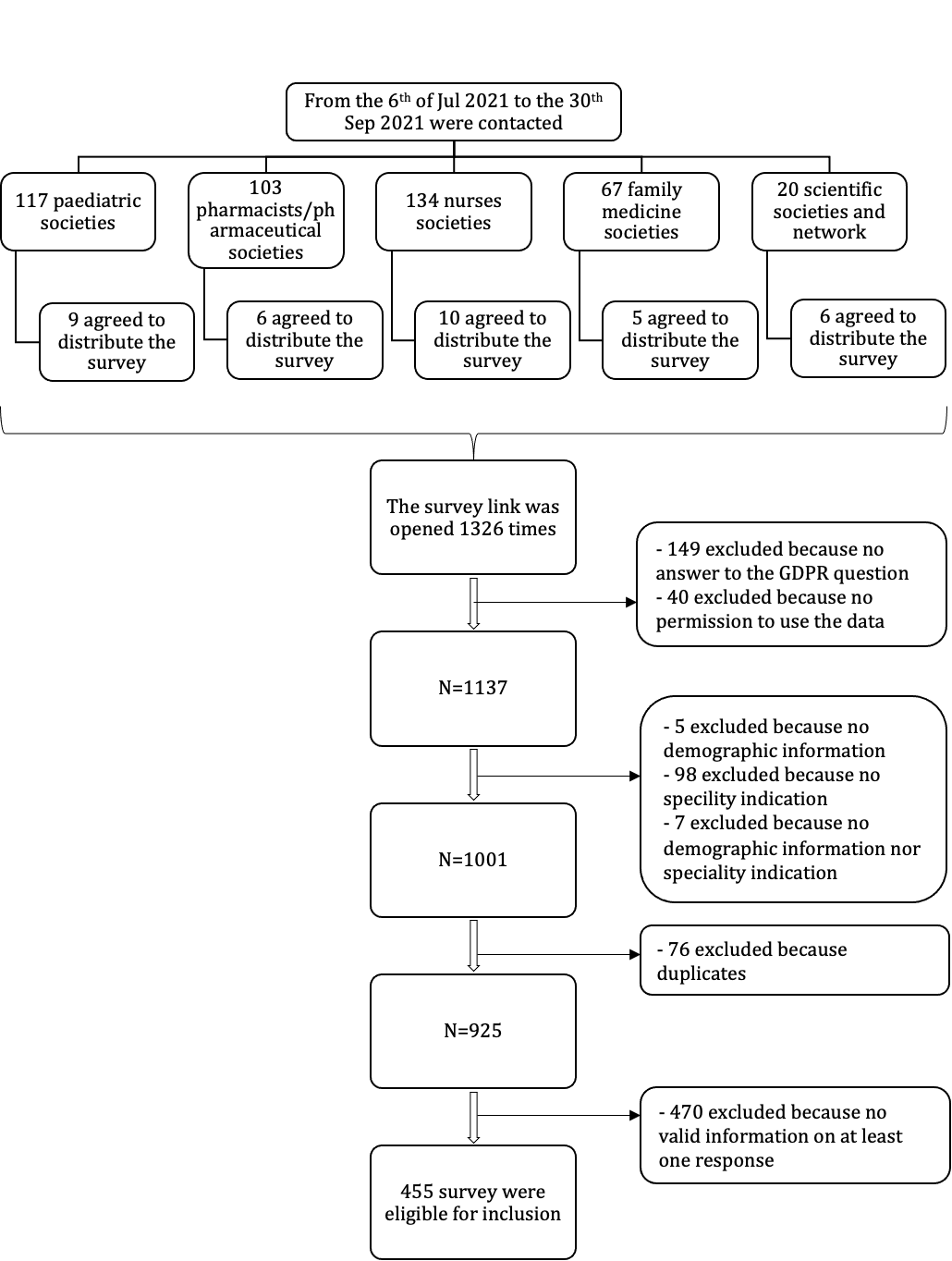


### Figure S2. Map of the countries where the respondents practice weighted by the number of medicines mentioned in the survey (panel A) and map of the countries where physicians (panel B), nurses (panel C) and pharmacists (panel D) responding to the survey practice.

**
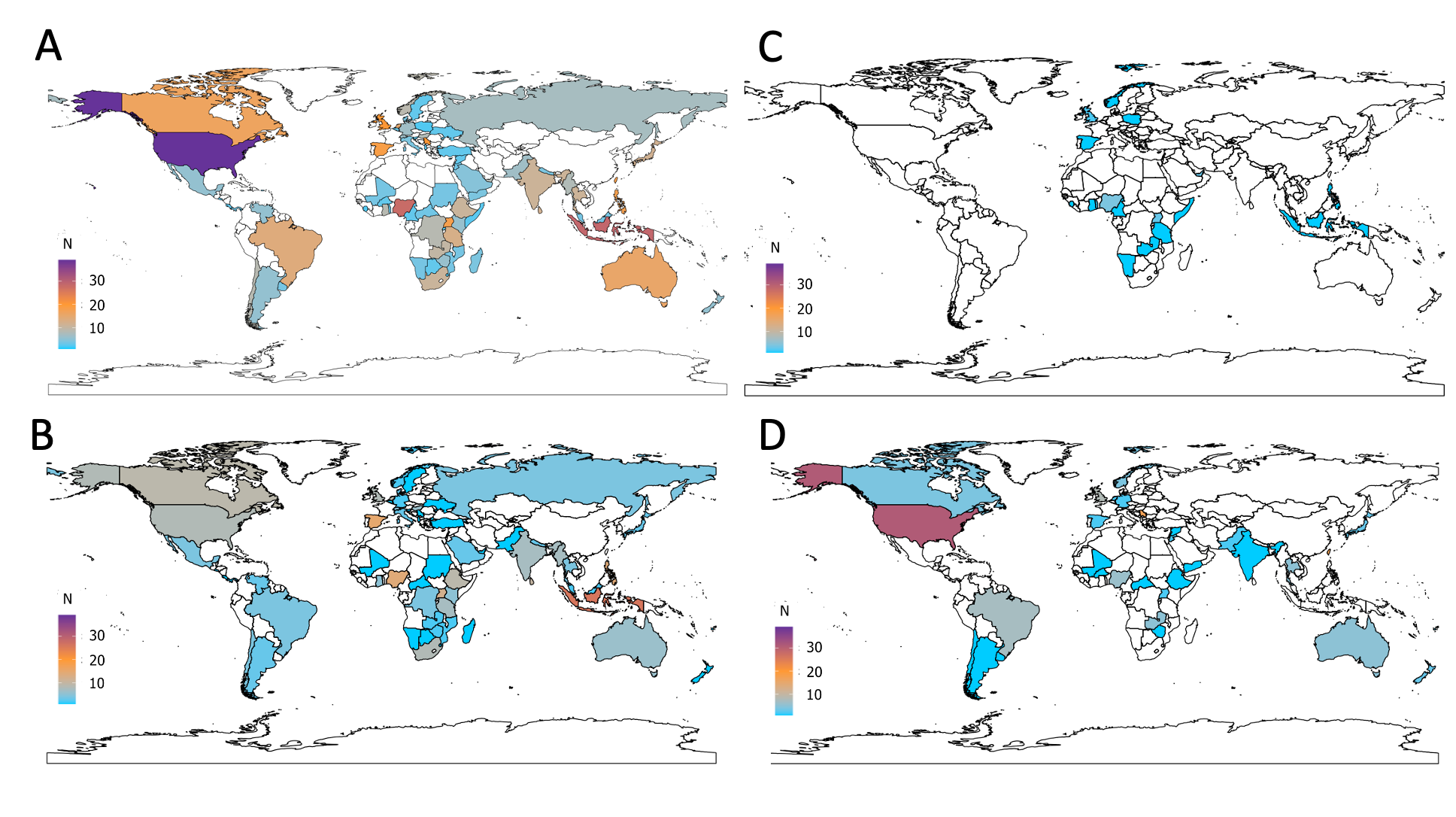
**

### Table S1. Distribution of the sub-specialties and the therapeutic classes of the medicines of the physicians, nurses, and pharmacists reporting medicines as problematic.

|  | **Hospital** | | | **Primary Care** | | | **Overall** | | |
| --- | --- | --- | --- | --- | --- | --- | --- | --- | --- |
|  | **Physician** | **Nurse** | **Pharmacist** | **Physician** | **Nurse** | **Pharmacist** | **Physician** | **Nurse** | **Pharmacist** |
|  | **(N=237)** | **(N=21)** | **(N=112)** | **(N=48)** | **(N=7)** | **(N=30)** | **(N=285)** | **(N=28)** | **(N=142)** |
|  | **N (%)** | **N (%)** | **N (%)** | **N (%)** | **N (%)** | **N (%)** | **N (%)** | **N (%)** | **N (%)** |
| **Sub-specialties** |  |  |  |  |  |  |  |  |  |
| Allergy and immunology | 15 (6) | 1 (5) | 10 (9) | - | - | 4 (13) | 15 (5) | 1 (4) | 14 (10) |
| Cardiology | 10 (4) | 1 (5) | 14 (13) | - | - | 4 (13) | 10 (4) | 1 (4) | 18 (13) |
| Dermatology | 3 (1) | - | 5 (5) | - | - | 5 (17) | 3 (1) | - | 10 (7) |
| Ear nose throat specialist | 1 (0) | - | 6 (5) | - | - | 2 (7) | 1 (0) | - | 8 (6) |
| Emergency medicine | 9 (4) | 6 (29) | 17 (15) | 3 (6) | - | - | 12 (4) | 6 (21) | 17 (12) |
| Endocrinology | 9 (4) | - | 11 (10) | 2 (4) | - | 5 (17) | 11 (4) | - | 16 (11) |
| Gastroenterology | 9 (4) | - | 12 (11) | - | - | 1 (3) | 9 (3) | - | 13 (9) |
| General paediatrics | 117 (49) | 7 (33) | 42 (38) | 24 (50) | 3 (43) | 7 (23) | 141 (50) | 10 (36) | 49 (35) |
| Infectious diseases | 61 (26) | 2 (10) | 24 (21) | 16 (33) | 1 (14) | 3 (10) | 77 (27) | 3 (11) | 27 (19) |
| Intensive care | 23 (10) | 5 (24) | 26 (23) | - | 1 (14) | 1 (3) | 23 (8) | 6 (21) | 27 (19) |
| Neonatology | 27 (11) | 4 (19) | 21 (19) | 3 (6) | 2 (29) | 2 (7) | 30 (11) | 6 (21) | 23 (16) |
| Nephrology | 8 (3) | 1 (5) | 10 (9) | - | - | 1 (3) | 8 (3) | 1 (4) | 11 (8) |
| Neurology | 12 (5) | 1 (5) | 6 (5) | 3 (6) | - | 1 (3) | 15 (5) | 1 (4) | 7 (5) |
| Nutrition | 8 (3) | 2 (10) | 8 (7) | 4 (8) | 4 (57) | 5 (17) | 12 (4) | 6 (21) | 13 (9) |
| Onco haematology | 10 (4) | - | 23 (21) | - | - | 2 (7) | 10 (4) | - | 25 (18) |
| Psychiatry | 3 (1) | - | 4 (4) | 2 (4) | - | 2 (7) | 5 (2) | - | 6 (4) |
| Pulmonology | 10 (4) | 1 (5) | 5 (5) | 1 (2) | - | 1 (3) | 11 (4) | 1 (4) | 6 (4) |
| Surgery | 4 (2) | 2 (10) | 8 (7) | 1 (2) | - | - | 5 (2) | 2 (7) | 8 (6) |
| Other spec | 4 (1) | 4 (19) | 16 (13) | 4 (8) | - | 6 (20) | 8 (3) | 4 (14) | 22 (15) |
| Adolescent health | 1 (0) | - | - | - | - | - | 1 (0) | - | - |
| Pharmacology | 1 (0) | - | - | 1 (2) | - | - | 2 (1) | - | - |
| Public health | 1 (0) | - | - | 2 (4) | - | - | 3 (1) | - | - |
| Rheumatology | 4 (2) | - | - | - | - | - | 4 (1) | - | - |
| **Therapeutic classes of the medicines** |  |  |  |  |  |  |  |  |  |
| Anaesthetics preoperative medicines and medical gases | 19 (8) | 6 (29) | 21 (19) | 1 (2) | 1 (14) | 4 (13) | 20 (7) | 7 (25) | 25 (18) |
| Medicines for pain and palliative care | 82 (35) | 9 (43) | 78 (70) | 15 (31) | 4 (57) | 13 (43) | 97 (34) | 13 (46) | 91 (64) |
| Antiallergics and medicines used in anaphylaxis | 76 (32) | 7 (33) | 36 (32) | 20 (42) | 1 (14) | 9 (30) | 96 (34) | 8 (29) | 45 (32) |
| Antidotes and other substances used in poisonings | 22 (9) | 2 (10) | 15 (13) |  |  | 2 (7) | 22 (8) | 2 (7) | 17 (12) |
| Anticonvulsants antiepileptics | 101 (43) | 10 (48) | 62 (55) | 14 (29) | 1 (14) | 14 (47) | 115 (40) | 11 (39) | 76 (54) |
| Anti infective medicines | 182 (77) | 7 (33) | 92 (82) | 41 (85) | 2 (29) | 15 (50) | 223 (78) | 9 (32) | 107 (75) |
| *Antibiotics* | *175 (74)* | *6 (29)* | *87 (78)* | *38 (79)* | *2 (29)* | *15 (50)* | *213 (75)* | *8 (29)* | *102 (72)* |
| *Antileprosy medicines* | *7 (3)* | *1 (5)* | *2 (2)* |  |  | *2 (7)* | *7 (3)* | *1 (4)* | *4 (3)* |
| *Antituberculosis medicines* | *88 (37)* |  | *20 (18)* | *13 (27)* |  | *3 (10)* | *101 (35)* |  | *23 (16)* |
| *Antifungal medicines* | *99 (42)* | *3 (14)* | *56 (50)* | *19 (40)* | *2 (29)* | *9 (30)* | *118 (41)* | *5 (18)* | *65 (46)* |
| *Antivirals* | *113 (48)* | *4 (19)* | *57 (51)* | *18 (38)* |  | *7 (23)* | *131 (46)* | *4 (14)* | *64 (45)* |
| *Antiprotozoal medicines* | *60 (25)* | *1 (5)* | *8 (7)* | *13 (27)* | *1 (14)* | *6 (20)* | *73 (26)* | *2 (7)* | *14 (10)* |
| *Medicines for ectoparasitic infections* | *22 (9)* |  | *5 (5)* | *6 (13)* | *1 (14)* | *5 (17)* | *28 (10)* | *1 (4)* | *10 (7)* |
| Antimigraine medicines | 21 (9) |  | 18 (16) | 8 (17) |  | 7 (23) | 29 (10) |  | 25 (18) |
| Immunomodulators and antineoplastics | 40 (17) | 1 (5) | 41 (37) | 1 (2) |  | 5 (17) | 41 (14) | 1 (4) | 46 (32) |
| Medicines affecting the blood | 44 (19) | 2 (10) | 35 (31) | 4 (8) | 1 (14) | 3 (10) | 48 (17) | 3 (11) | 38 (27) |
| Blood products of human origin and plasma sustitutes | 73 (31) | 6 (29) | 23 (21) | 2 (4) |  | 1 (3) | 75 (26) | 6 (21) | 24 (17) |
| Cardiovascular medicines | 71 (30) | 6 (29) | 59 (53) | 10 (21) |  | 15 (50) | 81 (28) | 6 (21) | 74 (52) |
| Dermatological medicines topical | 72 (30) | 2 (10) | 32 (29) | 23 (48) | 2 (29) | 16 (53) | 95 (33) | 4 (14) | 48 (34) |
| Diagnostic agents | 24 (10) |  | 15 (13) | 5 (10) |  | 2 (7) | 29 (10) |  | 17 (12) |
| Disinfectants and antiseptics | 50 (21) | 3 (14) | 15 (13) | 9 (19) | 2 (29) | 6 (20) | 59 (21) | 5 (18) | 21 (15) |
| Diuretics | 83 (35) | 11 (52) | 61 (55) | 8 (17) |  | 9 (30) | 91 (32) | 11 (39) | 70 (49) |
| Gastrointestinal medicines | 87 (37) | 6 (29) | 64 (57) | 17 (35) | 2 (29) | 14 (47) | 104 (37) | 8 (29) | 78 (55) |
| Medicines for endocrine disorders | 40 (17) | 1 (5) | 45 (40) | 8 (17) |  | 11 (37) | 48 (17) | 1 (4) | 56 (39) |
| Immunologicals | 48 (20) | 1 (5) | 22 (20) | 3 (6) |  | 4 (13) | 51 (18) | 1 (4) | 26 (18) |
| Muscle relaxants peripherally acting and cholinesterase inhibitors | 33 (14) | 3 (14) | 27 (24) | 1 (2) |  | 6 (20) | 34 (12) | 3 (11) | 33 (23) |
| Ophthalmological preparations | 29 (12) | 4 (19) | 25 (22) | 8 (17) | 1 (14) | 5 (17) | 37 (13) | 5 (18) | 30 (21) |
| Medicines for perinatal care | 49 (21) | 2 (10) | 24 (21) | 9 (19) | 2 (29) | 4 (13) | 58 (20) | 4 (14) | 28 (20) |
| Peritoneal dialysis solution | 20 (8) |  | 16 (14) |  |  | 2 (7) | 20 (7) |  | 18 (13) |
| Medicines for mental and behavioural disorders | 27 (11) | 3 (14) | 32 (29) | 10 (21) | 1 (14) | 8 (27) | 37 (13) | 4 (14) | 40 (28) |
| Medicines acting on the respiratory tract | 99 (42) | 8 (38) | 64 (57) | 25 (52) | 2 (29) | 12 (40) | 124 (44) | 10 (36) | 76 (54) |
| Solutions correcting water electrolyte and acid as disturbances | 97 (41) | 5 (24) | 58 (52) | 17 (35) |  | 9 (30) | 114 (40) | 5 (18) | 67 (47) |
| Vitamins and minerals | 116 (49) | 9 (43) | 53 (47) | 23 (48) | 5 (71) | 20 (67) | 139 (49) | 14 (50) | 73 (51) |
| Ear nose and throat medicines | 67 (28) | 4 (19) | 36 (32) | 17 (35) | 2 (29) | 13 (43) | 84 (30) | 6 (21) | 49 (35) |
| Medicines for diseases of joints | 43 (18) | 2 (10) | 23 (21) | 6 (13) | 1 (14) | 8 (27) | 49 (17) | 3 (11) | 31 (22) |

### Table S2. Missing products for paediatric care according to physicians, nurses, and pharmacists.

In the Excel file

### Table S3. Missing products for paediatric care according to physicians, nurses, and pharmacists by pharmaceutical form.

In the Excel file

### Table S4. Class of products considered problematic.

| **Class** | **Count of N** | % | **Sum of N** | % |
| --- | --- | --- | --- | --- |
| Antiinfective - antibacterials | 123 | 20·2 | 413 | 26·8 |
| Cardiovascular medicines | 54 | 8·9 | 170 | 11·0 |
| Antinfective - antivirals | 53 | 8·7 | 143 | 9·3 |
| Anticonvulsants/antiepileptics | 32 | 5·3 | 85 | 5·5 |
| Immunomodulators and antineoplastics | 47 | 7·7 | 81 | 5·3 |
| Medicines for pain and palliative care | 32 | 5·3 | 75 | 4·9 |
| Gastrointestinal medicines | 17 | 2·8 | 65 | 4·2 |
| Medicine acting on the respiratory tract | 23 | 3·8 | 59 | 3·8 |
| Medicines affecting the blood | 21 | 3·4 | 54 | 3·5 |
| Antinfective - antiprotozoal | 17 | 2·8 | 38 | 2·5 |
| Medicines for endocrine disorders | 13 | 2·1 | 33 | 2·1 |
| Immunomodulators for non-malignant disease/Antiallergics and medicines used in anaphylaxis | 5 | 0·8 | 30 | 1·9 |
| Antinfective - antifungal | 12 | 2·0 | 28 | 1·8 |
| Other | 21 | 3·4 | 27 | 1·8 |
| Anaesthetics, preoperative medicines and medical gases | 11 | 1·8 | 26 | 1·7 |
| Hormones and antihormones/Antiallergics and medicines used in anaphylaxis | 9 | 1·5 | 25 | 1·6 |
| Vitamins and minerals | 16 | 2·6 | 22 | 1·4 |
| Medicines for mental and behavioural disorders | 13 | 2·1 | 22 | 1·4 |
| Dermatological medicines | 10 | 1·6 | 17 | 1·1 |
| Medicines for reproductive health and perinatal care | 7 | 1·1 | 17 | 1·1 |
| Antiinfective - anthelminthics | 7 | 1·1 | 16 | 1·0 |
| Immunologicals | 10 | 1·6 | 14 | 0·9 |
| Antimigraine medicines | 4 | 0·7 | 13 | 0·8 |
| Antiallergics and medicines used in anaphylaxis | 9 | 1·5 | 12 | 0·8 |
| Immunomodulators and antineoplastics/Medicines for disease of joints | 3 | 0·5 | 11 | 0·7 |
| Antiinfective - antiprotozoal | 6 | 1·0 | 8 | 0·5 |
| Medicines for disease of joints | 5 | 0·8 | 8 | 0·5 |
| Solutions correcting water, electrolyte and acid-base disturbances | 7 | 1·1 | 7 | 0·5 |
| Antidotes and other substances used in poisonings | 4 | 0·7 | 4 | 0·3 |
| Antiseptics and disinfectants | 3 | 0·5 | 3 | 0·2 |
| Ophtalmological preparation | 3 | 0·5 | 3 | 0·2 |
| Blood products of human origin and plasma substitutes | 2 | 0·3 | 3 | 0·2 |
| Antidotes and other substances used in poisonings/Medicines acting on the respiratory tract | 2 | 0·3 | 2 | 0·1 |
| Medicine used in depressive disorders | 2 | 0·3 | 2 | 0·1 |
| Antiparkinsonism medicines | 1 | 0·2 | 1 | 0·1 |
| Diuretics | 1 | 0·2 | 1 | 0·1 |
| Ear, nose and throat medicines | 1 | 0·2 | 1 | 0·1 |
| Medicines used to treat gout | 1 | 0·2 | 1 | 0·1 |
| Muscle relaxants (peripherally-acting) and cholinesterase inhibitors | 1 | 0·2 | 1 | 0·1 |
| Peritoneal dialysis solution | 1 | 0·2 | 1 | 0·1 |
| **Grand Total** | **609** |  | **1542** |  |

### Table S5. Products considered most problematic for physicians, nurses, and pharmacists, stratified by pharmaceutical form [Legend: (*) Physicians, (**) Pharmacists, (^) Physicians and nurses, (^^) Physicians and pharmacists.]

In the Excel file
